# Heterogeneity in Disulfide Bond Reduction in IgG1 Antibodies Is Governed by Solvent Accessibility of the Cysteines

**DOI:** 10.3390/antib12040083

**Published:** 2023-12-13

**Authors:** Ramakrishnan Natesan, Andrew B. Dykstra, Akash Banerjee, Neeraj J. Agrawal

**Affiliations:** 1 Amgen Inc., Process Development, 360 Binney St., Cambridge, MA 02141, USA; rnatesan@amgen.com (R.N.); abaner12@amgen.com (A.B.); 2 Amgen Inc., Process Development, Thousand Oaks, CA 91320, USA; adykstra@amgen.com

**Keywords:** peptide mapping, differential alkylation, disulfide bond, molecular dynamics, SASA

## Abstract

We studied unpaired cysteine levels and disulfide bond susceptibility in four different γ-immunoglobulin antibodies using liquid chromatography–mass spectrometry. Our choice of differential alkylating agents ensures that the differential peaks are non-overlapping, thus allowing us to accurately quantify free cysteine levels. For each cysteine residue, we observed no more than 5% to be unpaired, and the free cysteine levels across antibodies were slightly higher in those containing lambda light chains. Interchain and hinge residues were highly susceptible to reducing stresses and showed a 100–1000-fold higher rate of reduction compared to intrachain cysteines. Estimations of the solvent-accessible surface for individual cysteines in IgG1, using an implicit all-atom molecular dynamics simulation, show that interchain and hinge cysteines have >1000-fold higher solvent accessibility compared to intrachain cysteines. Further analyses show that solvent accessibility and the rate of reduction are linearly correlated. Our work clearly establishes the fact that a cysteine’s accessibility to the surrounding solvent is one of the primary determinants of its disulfide bond stability.

## 1. Introduction

Therapeutic monoclonal antibodies (mAbs) such as Immunoglobulin G subclass 1 (IgG1) antibodies are used for treating various human diseases. More than 65 mAbs have been approved by the U.S. FDA to date [1], and many more antibodies are being evaluated in various clinical trials. Antibody molecules are prone to multiple post-translational modifications, and a few of these modifications or attributes, termed critical quality attributes (CQA), impact drug product quality by impacting the product’s potency and/or safety. Hence, the identification, understanding, and control of these critical quality attributes is required to ensure a drug product’s quality. The identification of CQA is often pursued during the early antibody development phase, and then the understanding of these CQA evolves as the antibody progresses in clinical development. Broadly, CQAs can be classified into two categories: those that change over time during storage (e.g., aspartic acid isomerization in acidic formulation under room temperature storage) and those that are stable during normal storage (e.g., N-glycosylations in the Fc domain). The former class of CQAs are generally controlled by process optimization, while the latter attributes are generally controlled by formulation and storage condition optimization.

**Figure 1:**
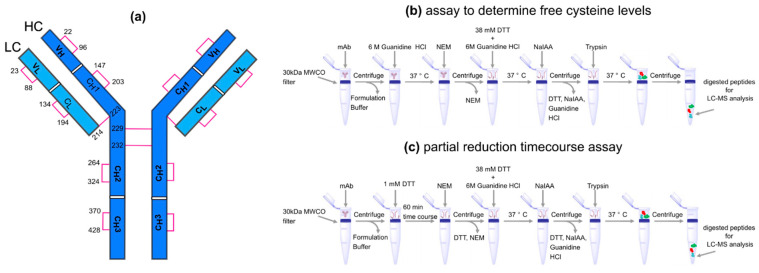
(**a**) An illustration of the interchain, intrachain, and hinge–hinge disulfide bonds for the standard cysteines in mAb1. The mapping between the shown cysteine residues to mAb2, mAb3, and mAb4 are shown in Supplementary Table S1. (**b**) Description of the assay to determine free cysteine levels in native and denatured conditions. (**c**) Description of the partial reduction time course assay.

Cysteine (Cys) residues in both the light chains (LC) and heavy chains (HC) of an antibody form multiple disulfide bonds (S–S) that play an important role in defining the structure, stability, and function of antibodies [2]. A typical IgG1 antibody has 4 interchain and 12 intrachain disulfide bonds, as illustrated in Figure 1a and Supplementary Table S1. In this study, we analyzed four different IgG1 mAbs, all of which are stable effector functionless (SEFL) antibodies that contain an additional S–S bond between two non-standard cysteine residues in the C_H_2 domain [3,4], see Supplementary Table S1. We used the mature linear numbering system to number the cysteines in each mAb molecule. The corresponding positions in the IMGT [5] and Kabat schemes [6] for the variable domain and in the EU numbering scheme [7] for the whole mAb may be found in Supplementary Table S1. Both inter- and intra-chain disulfide bonds play a dominant role in maintaining the properly folded secondary structure of an antibody, and hence, by extension, also govern antibody function. Lacy et al. [8] have shown that the thermal stability of mAbs is inversely correlated with the molar fraction of free cysteines. S–S bonds have low dissociation energies (~60 kcal/mol) and are hence more prone to *cleavage* when exposed to reducing agents or when subjected to stress conditions [9]. In addition to S–S bond cleavage, cysteine residues are also known to be highly susceptible to several other modifications including disulfide scrambling, racemization, cysteinylation, bridging to additional LC, and trisulfide formation [9,10], all of which could be CQAs that affect the binding, potency, pharmacokinetic profiles, and immunogenicity of the antibody. The presence of improper disulfide bond profiles during manufacturing [11,12] might increase molecular heterogeneity, impact the potency of the molecule [2], and affect manufacturing process yield [13].

Unpaired cysteine residues or free sulfhydryl groups are primarily a result of (a) incomplete disulfide bond formation during antibody synthesis/assembly and (b) the reductive cleavage of existing disulfide bonds. State-of-the-art methods for free cysteine characterization primarily use liquid chromatography–mass spectrometry (LC–MS) and liquid chromatography with tandem mass spectrometry (LC–MS/MS) methods to detect, locate, and quantify the levels of free thiols. In these methods, the free thiol groups in the mAbs are first alkylated with a suitable stable alkylating agent, followed by the reduction and labeling of the reduced thiols with another alkylating agent. The ideal differential alkylating agent should be such that it can sufficiently shift the spectrum to distinguish peaks eluted by free cysteines. For peptide mapping, ^12^C-Iodoacetic acid (IAA)/^13^C-IAA (+2 Da shift in cysteine mass) [14,15], d_0_-N-ethylmaleimide (NEM)/d_5_-NEM (+5 Da shift in cysteine mass) [16], and Iodoacetamide (IAM)/NEM (+60 Da shift in cysteine mass) [10,17] have been used as differential alkylating agents to quantify free cysteine levels in mAbs. For more details, we refer the reader to an excellent review of this topic [18]. For IgG1 antibodies, reports based on spectroscopic and mass spectrometric methods have reported the abundance of free sulfhydryl states on HC:C22 [10,19], HC:C96 [10,19], HC:C146 [10], HC:C202 [10], HC:C369 [10], HC:C42 [10], and LC:C22 [16]. These methods, however, do not provide information on the susceptibility of existing S–S bonds in response to environmental stresses.

Liu et al. [14] addressed this shortcoming by combining liquid chromatography–mass spectrometry (LC–MS) with differential alkylation to study disulfide bond susceptibility in native and reducing environments. The authors demonstrated that inter-chain disulfide bonds are more susceptible to reduction than intra-chain disulfide bonds, even in native environments. They also demonstrated that the L–H interchain disulfide bonds were more susceptible to reduction than the H–H interchain disulfide bonds. In the study by Liu et al. [14], the authors used ^12^C-iodoacetic acid to differentially alkylate cysteine residues following partial reduction and ^13^C-iodoacetic acid following denaturation/full reduction, prior to LC–MS analysis. The intensity of the monoisotopic peak corresponding to a peptide labeled with IAA was then compared to the intensity of ^13^C-IAA to determine the percent of reduction in the cysteine-containing peptide. However, the monoisotopic peak of a peptide labeled with ^13^C-IAA was isobaric with the second isotope (^13^C) of a peptide labeled with IAA, and a correction factor was needed by the authors to account for the overlapping isotopes of the two species. This becomes particularly problematic with larger peptides, wherein the monoisotopic peak is not the most intense peak, and the authors acknowledged that partial reduction at some residues was not evaluated because “the *m*/*z* (mass-to-charge ratio) corresponding to the monoisotopic molecular weight was too low for an accurate calculation”.

In this work, we improved on this approach by using NEM and sodium iodoacetate (NaIAA) to alkylate free cysteine residues. The mass of a single cysteine-containing peptide labeled with NEM increases by +125 Da, while that of a single cysteine-containing peptide labeled with NaIAA only increases by +67 Da, leading to significant mass shift (+58 Da) and well-resolved non-overlapping peaks. This method eliminated the need to correct for overlapping isotopes, and peak areas resulting from extracted ion chromatograms (two charge states per peptide, three isotopes per charge state) of the alkylated species were used to quantitate levels of each alkylated species. Because the monoisotopic *m*/*z* was not necessary for these calculations, this method also allowed for an evaluation of a wider range of cysteine-containing peptides. It should be noted that NaIAA/NEM induces a similar mass shift as the IAM/NEM previously used by Li et al. [10]. We chose NaIAA, since it showed a higher alkylation efficiency (lower % unlabeled cysteine) compared to IAM. The experimental protocol is depicted in Figure 1b,c. We also performed and analyzed the dynamics of full-length IgG1 antibodies to gain insight into how the physical and chemical environments could play a role in disulfide bond reduction.

## 2. Materials and Methods

All the reagents were of the highest purity. Tris-HCl was obtained from Teknova (Hollister, CA, USA), guanidine-HCl and dithiothreitol (DTT) in the no-weigh format were obtained from ThermoFisher Scientific (Waltham, MA, USA), and N-ethylmaleimide (NEM) and sodium iodoacetate (NaIAA) were obtained from Sigma-Aldrich (St. Louis, MO, USA). The purity of N-ethylmaleimide was ≥99.0% (BioUltra), and the reagent was stored at the vendor’s recommended temperature (2–8 °C) and protected from light. The purity of sodium iodoacetate was ≥99.5% (BioUltra), and the reagent was stored at the vendor’s recommended temperature (−20 °C) and protected from light. The purity of dithiothreitol (No-Weigh format) was not readily available, and the reagent was stored at the vendor’s recommended temperature (4 °C) and protected from light. All the solutions were made fresh immediately before experiments.

Biological therapeutics were captured on and subsequently prepared on Microcon 30 kDa molecular weight cut-off filters (MWCO) (MilliporeSigma, St. Louis, MO, USA). All the mAbs were generated recombinantly by Amgen using mammalian expression systems. These mAbs were then chromatographically purified.

### 2.1. Controls

For each mAb, three different controls representing different experimental conditions were prepared.

#### 2.1.1. Control 1, Representing Fully Reduced, Denatured Conditions

No DTT or NEM was added to the sample prior to full denaturation, reduction, and alkylation with NaIAA. This control allowed for the confident identification of IAA-labeled peptides, and because no peptides were labeled with NEM, this control also served to eliminate false positive NEM-labeled peptides.

#### 2.1.2. Control 2, Representing Native Conditions

No DTT was added to the sample prior to alkylation with NEM. Control 2 was used to determine the abundance of free cysteines in an intact mAb.

#### 2.1.3. Control 3, Representing Denatured Conditions

Samples were denatured and fully reduced by the addition of 100 μL of a solution containing 38 mM DTT, 6 M guanidine-HCl, 200 mM tris, pH 7.2, and incubation at 37 °C for 1 h, prior to alkylation with NEM. Retention times derived from this control allowed for the confident identification of low-level NEM-labeled peptides in partial reduction experiments.

#### 2.1.4. Disulfide Reduction Kinetics in DTT

A 100-µg sample was added to a 30 kDa MWCO filter, followed by centrifugation at 14,000× *g* with an Eppendorf 5430 centrifuge (Hamburg, Germany) to remove formulation buffer. The samples were washed by the addition of 200 µL 200 mM tris, pH 7.2, followed by centrifugation at 14,000× *g*. A total of 100 µL 1 mM DTT, 200 mM tris, pH 7.2 was added to each sample, followed by incubation at room temperature for 0 min, 1 min, 5 min, 10 min, 15 min, 30 min, and 60 min, respectively. This was followed by the addition of 100 µL 5 mM NEM, 200 mM tris, pH 7.2 and incubation in the dark at room temperature for 15 min. The samples were then centrifuged for 15 min at 14,000× *g* to remove the NEM alkylation solution (Figure 1c). The samples were washed by the addition of 200 µL 200 mM tris, pH 7.2 followed by centrifugation at 14,000× *g* and enzymatic digestion. 

#### 2.1.5. Reduced Disulfide Levels in the Absence of DTT

For each molecule, a sample was prepared in which the sample was denatured without DTT to monitor site-specific free cysteine levels. A 100-µg sample was added to a 30 kDa MWCO filter, followed by centrifugation at 14,000× *g* to remove formulation buffer. The samples were washed by the addition of 200 µL 6 M guanidine-HCl, 200 mM tris, pH 7.2 followed by centrifugation at 14,000× *g*. A total of 100 µL 6 M guanidine-HCl, 200 mM tris, pH 7.2 was added to each sample, followed by incubation at 37 °C for 1 h. The samples were centrifuged for 15 min at 14,000× *g* to remove denaturing buffer, followed by the addition of 100 µL 5 mM NEM, 6 M guanidine-HCl, 200 mM tris, pH 7.2 and incubated in the dark at room temperature for 15 min. Each sample was then centrifuged for 15 min at 14,000× *g* to remove the NEM alkylation solution. The samples were washed by the addition of 200 µL 200 mM tris, pH 7.2 followed by centrifugation at 14,000× *g* (Figure 1b) and enzymatic digestion. 

For preparing control 3, post-washing, 100 µL of a solution containing 38 mM DTT, 6 M guanidine-HCl, 200 mM tris, pH 7.2 was added to each sample, followed by incubation at 37 °C for 1 h. After 1 h, 100 µL 88 mM NEM, 6 M guanidine-HCl, 200 mM tris, pH 7.2 was added, followed by incubation in the dark at room temperature for 15 min. Each sample was then centrifuged for 15 min at 14,000× *g* to remove the NEM alkylation solution. The samples were washed by the addition of 200 µL 200 mM tris, pH 7.2 followed by centrifugation at 14,000× *g* and enzymatic digestion.

#### 2.1.6. Enzymatic Digestion

Each of the samples above were digested using a method adapted from [20]. A 37-µL denaturing solution (6 M guanidine-HCl, 200 mM tris, 20 mM methionine, pH 8.3) and 3 µL 500 mM DTT was added to each sample, followed by incubation at 37 °C for 30 min. After denaturing and reducing, the samples were alkylated by the addition of 33 µL of the denaturing solution and 7 µL 500 mM NaIAA and incubation at room temperature in the dark for 20 min. Excess alkylating reagent was quenched by adding 34 µL of the denaturing solution and 6 µL 500 mM DTT. The samples were centrifuged at 14,000× *g* to remove denaturing, reducing, and alkylating reagents, followed by three cycles of adding 200 µL of a digest solution (50 mM tris, 20 mM methionine, pH 7.2) and centrifuging at 14,000× *g*. The samples were digested by adding 35 µL of the digest solution and 5 µL 1 mg/mL trypsin and incubating at 37 °C for 60 min. The samples were centrifuged 14,000× *g*, and the digested peptides were collected for LC–MS analysis.

#### 2.1.7. Mass Spectrometry

The digested samples were analyzed using a Thermo Scientific U-3000 UPLC system connected in-line to a Thermo Scientific Q-Exactive (Waltham, MA, USA). A reversed phase HPLC column, Agilent ZORBAX Rapid Resolution HD StableBond, C18, 2.1 mm × 150 mm, 1.8 μm (Santa Clara, CA, USA) was used to separate the peptides with the column temperature at 50 °C and mobile phase A: 0.1% formic acid (*v*/*v*) in water and mobile phase B: 0.1% formic acid in acetonitrile. The gradient (hold %B at 1.0% for 10 min, then 1–10% B for 1 min and 10–36% B for 67 min) was performed at 0.25 mL/min. About 3 µg of each sample was injected. The mass spectrometer operated in positive mode, using data-dependent acquisition (top 5). The MS1 resolution was set to 35,000, with a scan range of 200–2000 *m*/*z*. MS1 automatic gain control was set to 1 × 10^6^, and maximum IT was set to 200 ms. MS2 were acquired at 17,500 resolution, using a stepped normalized collision energy of 22, 25, and 28, with automatic gain control set to 5 × 10^5^. 

### 2.2. Data Analysis

Peptide identification was performed using MassAnalyzer, version 3.02 [21]. Relative quantitation was performed using Skyline, version 3.7.0.11317 [22]. For each molecule, a workbook was created containing NEM-alkylated (+125.047679 Da) and NaIAA-alkylated (+58.005479 Da) versions of all cysteine-containing peptides. Unalkylated versions of each peptide were also monitored, but the signal for these was negligible. The total area of alkylated peptides was determined by extracting ion chromatograms from two charge states per peptide (three isotopes per charge state). Mass accuracy for each peptide charge state was better than 5 ppm. For NEM-alkylated peptides, the isotopic dot product for each charge state was better than 0.9 in the control sample that was denatured and reduced prior to alkylation with NEM; for NaIAA-alkylated peptides, the isotopic dot product for each charge state was better than 0.9 in the control sample in which NEM was not added. NEM alkylation levels were calculated by dividing the total area of the NEM-alkylated peptide by the sum of total areas from the NEM-alkylated and NaIAA-alkylated peptides. It should be noted that both hinge cysteine residues (C226, C229) were found on the same tryptic peptide conserved among all four molecules analyzed. Four total peaks corresponding to this peptide were monitored: one peak with both cysteine residues alkylated with NaIAA; one peak with both cysteine residues alkylated with NEM; and two peaks containing one NEM-alkylated residue and one NaIAA-alkylated residue. Total hinge NEM-alkylation levels were calculated by summing both the peak area of the three species containing alkylated NEM and normalization by the peak area summed from all four peaks.

#### 2.2.1. Selection of Alkylating Reagents

The complete enzymatic digestion of mAbs requires the full denaturation and reduction of disulfide bonds to expose all residues susceptible to digestion. Reduced cysteine residues must then be alkylated to prevent disulfide reformation and disulfide scrambling, which may result in incomplete digestion. To monitor disulfide stability, multiple alkylating reagents (NEM and NaIAA) were used for alkylation. Iodoacetamide (IAM) was initially considered as a second alkylating reagent to use in conjunction with NaIAA, but this reagent was ultimately not used because the mass shift induced by IAM differs from that induced by NaIAA by only 1 Da, which may complicate quantitation due to overlapping isotopic peaks. In these experiments, mAbs were incubated with NEM to alkylate cysteine residues following a partial reduction time course in the absence of denaturant, while NaIAA was used to alkylate cysteine residues, only after the removal of free NEM and after full denaturation and reduction by incubating for 30 min in 6 M guanidine HCl, 38 mM DTT at 37 °C. Because alkylation with NEM and NaIAA induces mass shifts on cysteine residues that are easily differentiated with mass spectrometry (+125.047679 Da vs. +58.005479 Da), this method distinguishes disulfides that are reduced during the partial reduction time course from those that are reduced during the sample preparation for digestion. Higher levels of NEM alkylation suggest that the corresponding cysteine residues are more susceptible to reduction during the partial reduction time course. 

#### 2.2.2. Selection of Controls

Three control samples for each mAb were prepared for these experiments. While preparing the first control (control 1), no DTT or NEM was added to the sample prior to full denaturation, reduction, and NaIAA alkylation. This first control provided information on the background while extracting ion chromatograms of the cysteine-containing peptides. While preparing the second control (control 2), no DTT was added to the sample prior to alkylation with NEM. This control allowed for the monitoring of (most likely surface-exposed) reduced cysteine levels. The third control (control 3) was denatured and fully reduced prior to alkylation with NEM. Following time course experiments described below, NEM-alkylation levels for many of the cysteine-containing peptides were low. Since the NEM alkylation levels of this third control were near complete, this control allowed for a more confident identification and monitoring of NEM-alkylation levels.

#### 2.2.3. Selection of DTT Concentration for Partial Reduction

To probe disulfide bond stability to the reducing conditions, mAbs were incubated with a relatively low concentration of DTT in the absence of denaturant to induce a partial reduction of disulfide bonds. Initial experiments were performed to optimize the DTT concentration; if the DTT concentration was too high, disulfide reduction would occur too fast to reliably determine reduction rates. It was observed that, after incubation with 1 mM DTT for 60 min, hinge cysteine residues for the mAbs ranged from 72–87% alkylated with NEM. These results were compared to the alkylation levels, which ranged from 94–100%, of a fully denatured and reduced control (i.e., control 3). Since 1 mM DTT resulted in significant but incomplete reduction after incubation for 60 min at room temperature, further partial reduction experiments were performed using this DTT concentration.

#### 2.2.4. Molecular Dynamics Simulations

Homology models corresponding to full-length IgG1κ and IgG1λ mAbs were generated and prepared for molecular simulations as described in our earlier work; see [23]. We performed all molecular dynamics simulations with *OpenMM* [24] using AMBER *ff19SB* [25] force field for proteins. All the simulations were run in an NPT ensemble, with T = 300 K and P = 1 atmosphere. Hydrogen bonds were constrained allowing us to use a timestep *dt* = 2 fs. The temperature and pressure were regulated using a Monte Carlo Barostat, for which we chose a friction coupling of 1 ps^−1^, a Barostat interval of 25 × *dt*, and a constraint tolerance of 1 × 10^−6^. For implicit solvent simulations, we used the Hawkins–Cramer–Truhlar GBSA model [26], with a salt concentration of 150 mM. In all our simulations, the initial structure was first minimized, then equilibrated for 1 ns, and then followed by production runs. Typical runs of a full-length IgG1 mAb, with 20,502 atoms and on a single NVIDIA GTX 1080 GPU card, yielded 10–13 ns a day. We analyzed all simulation trajectories using MDTraj [27]. We employed the MDTraj implementation of Shrake–Rupley method [28] to compute the solvent-accessible surface area for the cysteine residues. 

## 3. Results

### 3.1. All the mAbs Show a <5% Abundance of Free Sulfhydryl Groups

**Figure 2:**
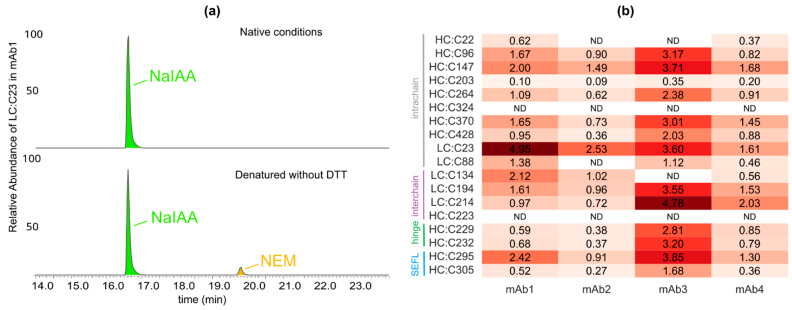
Free cysteine levels in native and denatured conditions. (**a**) Extracted ion chromatogram (EIC) for LC:C23 in mAb1, showing its intact (marked by NaIAA) and reduced (marked by NEM) levels in native and denatured conditions. (**b**) Comparison of free cysteine levels across the four mAbs in denatured conditions for the marked residues involved in intrachain, interchain, and hinge disulfide bonds. The non-standard SEFL residues shown alongside form intrachain S–S bonds. ND indicates that the value could not be determined.

We performed LC–MS/MS mapping of cysteine residues in four different IgG1 molecules representing a variety of germlines: (i) mAb1 (VK3/VH3), (ii) mAb2 (VK1/VH1), (iii) mAb3 (VL1/VH1), and (iv) mAb4 (VL2/VH2); see Supplementary Table S1. In addition to NaIAA- and NEM-labeled peptides, each peptide evaluated was also assessed for unlabeled cysteine residues. In each sample, the signal for an unlabeled peptide was negligible, hence ensuring that all the cysteines were tagged and accounted for. We first studied the levels of free cysteines in each of the mAbs by tagging free thiols with NEM in their native and denatured conditions, as described in the Materials and Methods section. For the latter, mAbs were first fully denatured by the addition of 6 M Guanidine HCl before the addition of NEM. We expected all natively reduced cysteines to be alkylated with NEM and all intact cysteines, which were subsequently reduced and denatured, to be alkylated with NaIAA. The results from our analysis are displayed in Figure 2.

The extracted ion chromatogram (EIC) for mAb1/LC:C23 shown in Figure 2a is an example of a cysteine residue that shows disulfide bond breakage in a population of the mAb sample. Our results show that ~5% of the mAb1 population contains a free thiol at LC:C23, as measured by the corresponding NEM signals. Interestingly, the NEM signals were only more prominent in the denatured conditions compared to native conditions, where we only observed <1% abundance of free LC:C23; see Figure S1. The difference observed in NEM signals between the native and denatured conditions is most probably related to the solvent accessibility of the cysteines, which in turn determines their access to the alkylating agent. In the native state, where the secondary structure of mAb1 is intact, LC:C23 has poor solvent accessibility, resulting in a very low probability of alkylation with NEM. Upon denaturation, its accessibility to NEM increases, resulting in an increased NEM signal. Similar differences between the native and denatured conditions have been reported in the past [19]. The role of accessibility can also be understood by comparing the abundance of the peripherally located hinge residues mAb1/HC:229 and mAb1/HC:232, whose solvent accessibility does not change upon denaturation. For such well-hydrated residues, we only observe a very slight increase in the NEM signals compared to that for the buried cysteines. The same hypothesis can also explain why LC:C88, the S–S bond pair for LC:C23, shows roughly four-fold lower free thiol levels compared to LC:C23, despite being in a reduced state. Previous studies have also reported similar phenomena and have hypothesized that a difference in solvent accessibility could play an important role in determining the alkylation levels of free thiols in intact mAbs [10]. It should be noted that a partial denaturation of the protein can influence access to the alkylating agent, which in turn can affect our estimates of the free sulfhydryl levels. For example, partial denaturation in native conditions can increase access to the alkylating agent, leading to a higher abundance, while the same in denaturing conditions can result in lower access to the alkylating agent, leading to lower-than-expected abundance.

Our results showed that LC:C23 has the highest free cysteine abundance levels in IgG1κ isotypes mAb1 and mAb2, both of which are composed of kappa light chains (Figure 2b). On the other hand, IgG1λ isotypes mAb3 and mAb4, both containing lambda light chains, showed a high abundance of free cysteines across multiple residues. These findings were consistent with similar observations reported earlier [14,29]. Shen et al. [29] showed that the presence of the terminal serine residue flanking LC:C214 in IgG1λ. variants leads to weaker interchain disulfide bonds. Our results indicate that free levels of LC:C214 in mAb3 and mAb4 are also much higher compared to those in the IgG1κ variants mAb1 and mAb2.

### 3.2. Interchain and Hinge–Hinge Disulfide Bonds Are More Susceptible to Reducing Stress

Having established the baseline abundance of free cysteines, we next studied the susceptibility of each cysteine residue to a reducing stress. The mAbs were first treated with 1 mM DTT for seven different exposure times (τ=0, 1, 5, 10, 15, 30, 60 min). The treated mAbs then contained a joint population of cysteines that were free before reduction and whose S–S bonds reduced in response to weak reducing stress. These free cysteines were tagged with NEM before full reduction and tagging with NaIAA.

**Figure 3:**
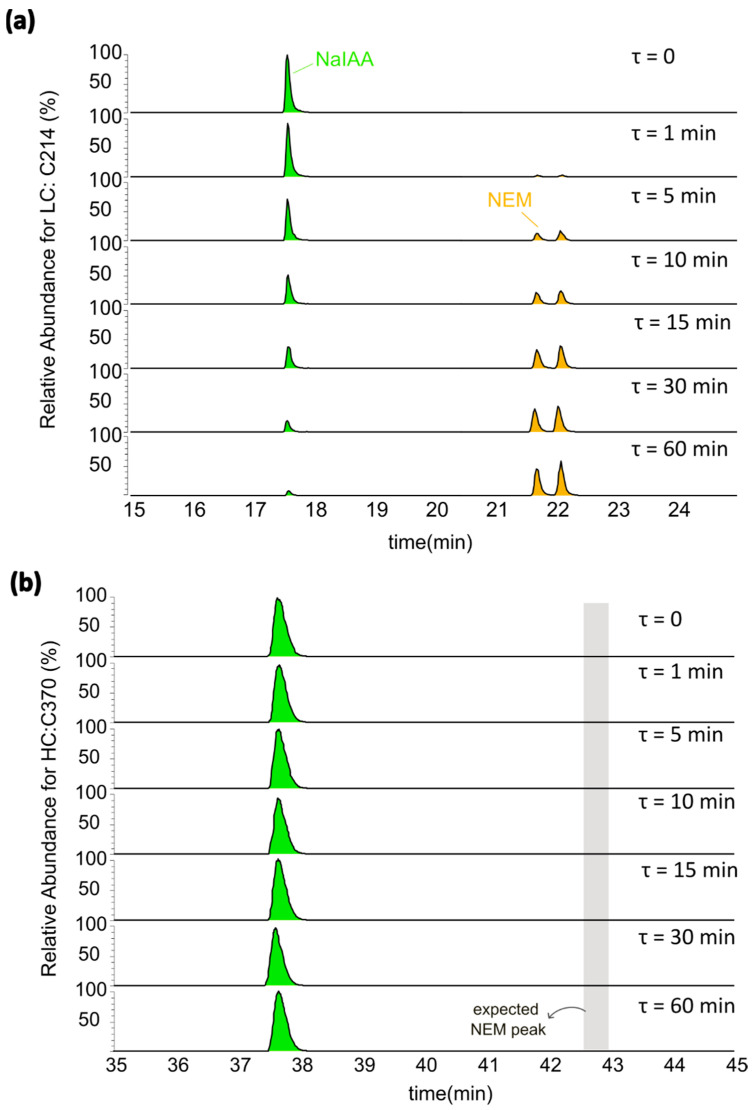
Extracted ion chromatograms for mAb4 showing the relative abundance of differentially alkylated cysteines in response to partial reduction with 1 mM DTT for seven different exposure times τ. (**a**) LC:C214 is more susceptible to reduction since its NaIAA-alkylated peaks become more abundant with an increase in the exposure time τ. (**b**) HC:C370 is the least susceptible to reduction since the abundance of NaIAA peaks are nearly zero, even after 60 min of exposure to a reducing agent. The NEM peak is expected to elute in the shaded region marked in the chromatogram.

**Figure 4:**
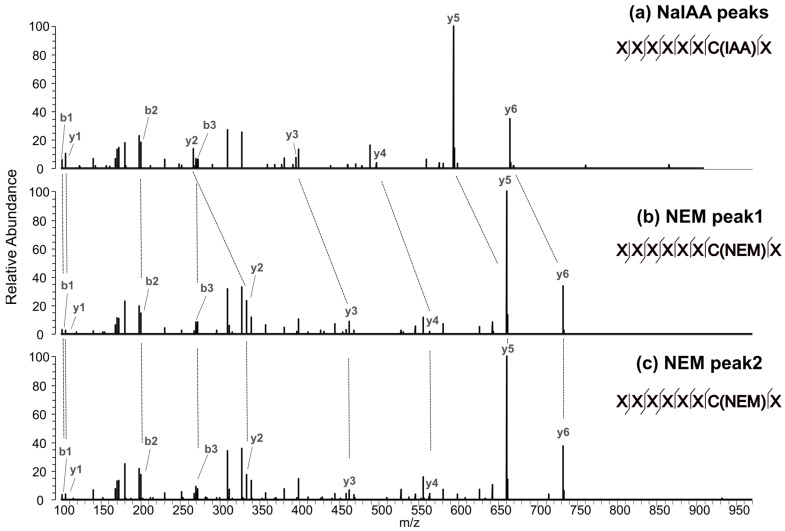
Tandem mass spectrometry (MSMS) spectra showing the relative abundance of cysteine-containing peptides alkylated with (**a**) NaIAA and (**b**,**c**) NEM. When alkylated with NEM, peaks y2–y6 are shifted to the right of the corresponding NaIAA peaks. Panels (**b**,**c**) corresponding to the observed double peaks resulting from diastereomer states show identical spectra.

The EICs corresponding to the time course for mAb4/LC:C214 and mAb4/HC:C370 are displayed in Figure 3. The chromatogram for mAb4/LC:C214 in Figure 3a illustrates how NEM-alkylated peptides, containing cysteine residues that are highly susceptible to reducing stresses, shift from a nearly undetectable NEM signal at τ=0 to being the primary species after incubation with 1 mM DTT for τ=60 min. On the other hand, Figure 3b shows that cysteine residues that are minimally susceptible to reduction only contain signals corresponding to NaIAA alkylation, and the NEM-alkylated species are not detectable. It should be noted that the double peaks for the NEM-alkylated species seen in Figure 3a were also observed for many of the peptides monitored in our experiments. These double peaks were likely observed due to the formation of diastereomers during alkylation with NEM, as previously reported [30]. MSMS confirmed that both peaks correspond to the peptide-containing mAb4/LC:C214 (Figure 4) were differentially shifted in response to alkylation with NEM or NaIAA, and the spectra for the double peaks were identical. For all the molecules, post incubation with 1 mM DTT for 60 min, we observed maximum NEM alkylation levels for (a) the hinge cysteine residues mAb4/HC:C229 and mAb4/HC:C232 and (b) the interchain disulfide bond cysteine mAb4/LC:C214. See Supplementary Table S1 for the corresponding residue numbers in other mAbs. The levels of the other interchain cysteine mAb4/HC:C220 could not be resolved in our experiments, since the tryptic peptide-containing mAb4/HC:C220 was a conserved four-residue peptide that is not retained on the columns. 

### 3.3. Estimation of the Cysteine-Specific Reduction Rate Constant

We modeled the kinetics of a cysteine residue reduction when exposed to DTT (where the levels of NEM alkylation are quantified as the levels of reduced cysteine) using the following pseudo first order kinetics [31]:

(1) C→CH. 

Here, C is the population of an oxidized (i.e., disulfide bonded) cysteine, and CH denotes the population of the same cysteine residue in its reduced state (i.e., NEM alkylated). We modeled the reduction kinetics as a first order rate equation:

(2) $$\frac{d\left[C\right]}{dt}=-k\left[C\right].\;$$

Here, $\left[C\right]$ is the molar concentration of a particular cysteine residue in its oxidized state, and k is the pseudo first order rate constant. The time dependent concentration of the oxidized state at a chosen time τ can then be expressed as:

(3) $${\left[C\right]}_{\tau }={\left[C\right]}_{0}\;e^{-k\tau }.$$

Noting that ${\left[C\right]}_{\tau }+{\left[CH\right]}_{\tau }={\left[C\right]}_{0}+{\left[CH\right]}_{0},$ and defining the fraction reduced at time τ as $F_{\tau }=\frac{{\left[CH\right]}_{\tau }}{{\left[C\right]}_{0}+{\left[CH\right]}_{0}}$, the equation above can be re-expressed as:

(4) $$\left(1-F_{\tau }\right)=\left(1-F_{0}\right)e^{-k\tau }$$

,

from which the rate constant k can be estimated as:

(5) $$k=\frac{1}{\tau }\left(\ln\left(1-F_{0}\right)-\ln\left(1-F_{\tau }\right)\right).$$

**Figure 5:**
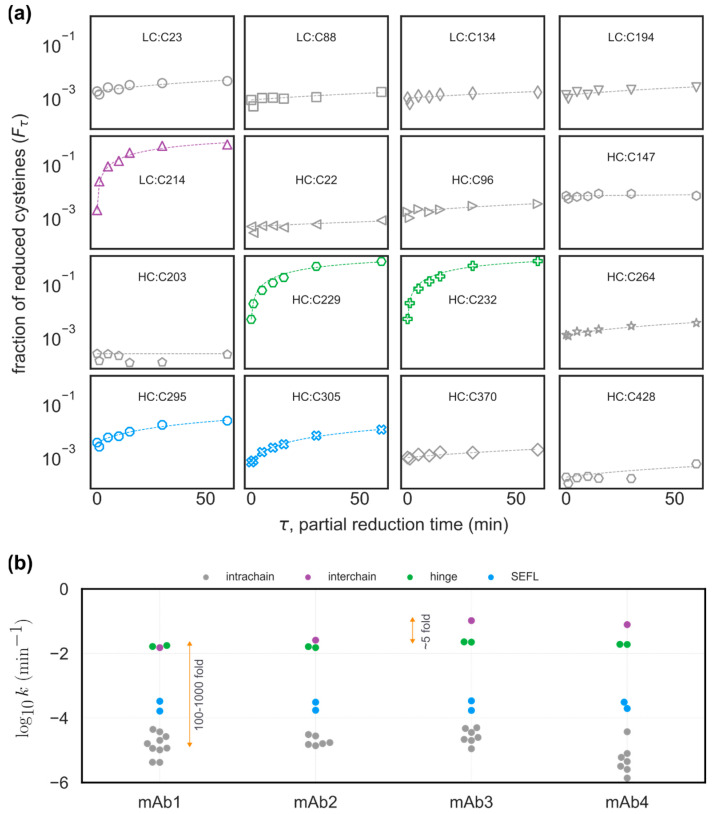
Cysteines involved in inter-chain and inter-hinge S–S bonds show higher susceptibility to reduction. (**a**) Time series plot showing the measured fraction of reduced cysteines $\left(F_{\tau }\right)$ in mAb1 partially reduced by exposure to 6M DTT without denaturation. Symbols denote experimental data, and dashed lines represent the best fit of the data to Equation (4). (**b**) The estimated values of reduction rate $\left(k\right)$ for all cysteines across the four mAbs are shown.

For each cysteine residue, the value of the reduction rate constant k was calculated by fitting the measured relative abundances of NEM-alkylated species to Equation (5). The timepoints τ in the experimental data correspond to the times of exposure to 1 mM DTT. In Figure 5a, symbols denote the experimentally measured NEM alkylation levels for all cysteine residues in mAb1, and the dashed lines represent the corresponding best fit determined using Equation (5). The residue-specific reduction rate constant k for all four mAbs is shown in Figure 5b, where we have compared the reduction rates across the four mAbs. From our analysis, we estimate that the interchain and hinge cysteines have ~100–1000-fold higher susceptibility to reduction compared to cysteines involved in intrachain disulfide bonds. We also observed that interchain cysteine residues in IgG1λ mAbs are ~5-fold more susceptible to reduction compared to their IgG1κ counterparts, as can clearly be seen in Figure 5b, as reported previously [29].

Qin et al. [32] have previously shown that the stability of a disulfide bond primarily depends on *(a) the distance between the sulfur atoms; (b) the orientations of the sulfur atoms* defined in terms of angles $\Theta_{1}$ and $\Theta_{2}$ shown in Supplementary Figure S3; and *(c) the solvent accessibility of the cysteine residues.* We examined the role of these physical mechanisms using molecular dynamics simulations of full-length IgG1κ (VK3/VH3 germline) and IgG1λ (VL3/VH3 germline) mAbs, performed in implicit solvent conditions as described in the Materials and Methods section and detailed in our earlier publications [23,33]. Briefly, we used Discovery Studio to generate homology models for each molecule, using the 1HZH structure in RCSB PDB [34] as the template. The structure with the lowest energy was then minimized and equilibrated, and the relaxed conformation was used as the starting configuration for implicit solvent simulations, as described in the Materials and Methods section. For each molecule, we performed the simulations in duplicate, each 500 ns long.

**Figure 6:**
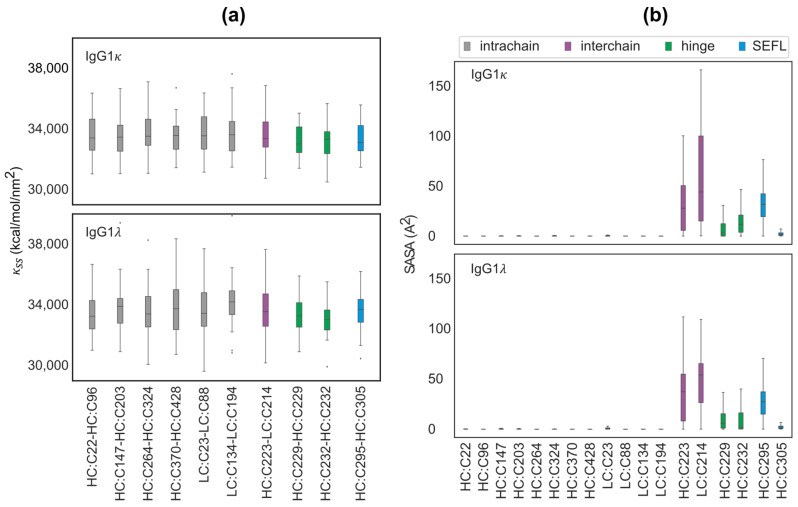
Analysis of S–S bond fluctuations in 500 ns implicit solvent molecular dynamics simulations (in duplicates) of IgG1κ and IgG1λ molecules. (**a**) $\kappa_{SS}$, the estimated S–S bond stiffness for various intrachain, interchain, hinge–hinge, and SEFL disulfide bonds, displayed as box plots, show a nearly identical distribution across all disulfide bonds. (**b**) Cysteine-specific solvent-accessible surface area measured over the entire trajectory shows nearly zero accessibility for intrachain cysteine residues and maximum accessibility for interchain cysteines.

We first studied the fluctuations in the S–S bond distances for the 16 canonical and the 2 SEFL disulfide bonds associated with full-length IgG1κ and IgG1λ molecules. The duplicate-averaged and normalized distributions for each disulfide bond in both molecules are shown in Supplementary Figure S2. Our analysis showed identical Boltzmann distributions for all S–S bonds, with an equilibrium bond length of 2.1 A. We computed the variance $\sigma^{2}$ for each disulfide bond by fitting the distribution to a normal function, from which we estimated the bond stiffness as $\kappa_{ss}=\frac{k_{B}T}{\sigma^{2}}$. Here, $k_{B}$ is the Boltzmann constant, and T is the absolute temperature taken to be 300 K. The estimates for $\kappa_{SS}$ for each S–S bond are displayed as a bond-specific boxplot in Figure 6a. We did not observe any noticeable difference in the values of $\kappa_{SS}$ between the various cysteine residues. Similarly, we also did not observe any noticeable difference in the orientations of the thiol groups; see Supplementary Figure S4. These findings strongly suggest that the experimentally observed variations in the rates of reduction could be primarily determined by the solvent accessibility of cysteines.

We next computed the solvent-accessible surface area (SASA) for each cysteine residue over the entire trajectory as described in the Materials and Methods section. The computed value of SASA represents the surface area of the protein accessible to water, which in turn sets the upper cutoff of the area that is accessible for a reducing agent. The residue-specific SASA shown in Figure 6b shows a clear delineation between cysteines. Our results indicate that all intrachain cysteines have nearly zero solvent accessibility, while all hinge, SEFL, and interchain residues have non-zero accessibility. Particularly, interchain cysteines (HC:C223 and LC:C214) are highly accessible to the solvent, which could potentially explain the high rate of reduction for these residues observed in Figure 5b. From a structure perspective, since the interchain, SEFL, and hinge residues are peripherally located, they tend to have higher SASA, leading to higher S–S bond susceptibility, while intrachain cysteines buried in the framework regions have small solvent accessibility, resulting in lower reduction in their disulfide bonds.

**Figure 7:**
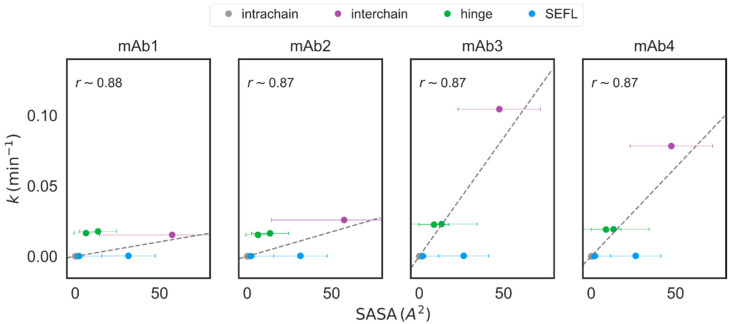
Averaged SASA measured in molecular dynamics simulations shows excellent correlation with the reduction rate constant k estimated from experiments. The best linear fit (excluding the data for hinge residues) is shown as dashed gray lines, and the corresponding Pearson correlation coefficient r for each mAb is shown alongside. The datapoints corresponding to intrachain, interchain, hinge, and SEFL cysteines are plotted using the same color scheme used in Figure 6.

We asked if the molecule-specific SASA value estimated from our simulations could explain the cysteine-specific differences in the reduction rate constant k by analyzing their correlation as displayed in Figure 7. k for mAb1 and mAb2 were compared to SASA for IgG1κ, while that for mAb3 and mAb4 was compared to SASA for IgG1λ. Cysteine-specific SASA showed strong linear correlation (Pearson *r* > 0.85 for all mAbs) with the experimentally measured values of k and confirms our hypothesis that disulfide bond susceptibility is strongly dependent on the solvent accessibility of the specific residues. A similar trend has been reported by Song et al. [35] in the study of inter-chain disulfide bond-reduced low molecular weight (LMW) IgG1 and IgG4 antibodies. They demonstrated that the LMW formation pathway is mainly determined by the strength of the interchain or the HC–HC disulfide bonds, which in turn depends on the solvent accessibility of the associated cysteines. Similarly, Li et al. [10] have reasoned that poor accessibility to buried intrachain cysteines could potentially explain their observations that these intrachain cysteines show negligible reduction, even when subject to high pH and elevated temperatures. We do observe some outliers that do not conform to this seemingly straightforward rule. Including the hinge residues in the correlation analysis significantly reduces the correlation coefficient, particularly for mAb1 and mAb2, for which we observe high reduction rates of hinge disulfide bonds, despite their much smaller solvent accessibility; see Supplementary Table S2. Whether the high flexibility of the hinge regions, which in turn could translate into additional stress on the hinge disulfide bonds, could explain this discrepancy is yet to be determined; see [9] for detailed discussions.

## 4. Discussions

Proper disulfide bridging in mAbs is a critical quality attribute that is known to affect structure, efficacy, and pharmacokinetics. A continuous monitoring of disulfide bond profiles during development, manufacturing, and storage is essential to ensure quality and guarantee molecules with maximum efficacy and safety profiles. We used LC–MS/MS to estimate the levels of free cysteines in four different IgG1 antibodies belonging to four different germlines. Free and intact cysteines were differentially alkylated with N-ethylmaleimide (NEM) and sodium iodoacetate (NaIAA), respectively. Our choice of alkylating agents, with a mass difference of 58 Da and high alkylation efficiency, has allowed us to better resolve peaks from differentially alkylated cysteine-containing peptides and accurately measure the population of free cysteines. We applied the same approach to map and quantify disulfide bonds that are highly susceptible to reducing stresses.

Table 1 shows a comparison of our results to the abundances of unpaired cysteines reported in the literature [10,14,16,19]. Our results confirm the consensus that intrachain cysteines are well shielded from the solvent, and, as a result, have lower levels of unpaired cysteines and display poor susceptibility to reduction by a reducing agent. The only exception to this rule is the high unpaired levels for LC:C88 reported by Robotham and Kelly [16]. We believe that this significant difference could be due to (a) their method of using two differentially alkylated populations of mAbs to estimate free sulfhydryl abundance and (b) their choice of mAbs, which is a strong determinant of unpaired cysteine levels. Despite the vast heterogeneity in the data shown in Table 1, it can be estimated, with high confidence, that the abundance of free sulfhydryl levels in mAbs is below 5%.

Our analysis shows that antibody synthesis systems are very robust and have tight quality controls, as evidenced by the less than 5% error in disulfide bridging in all cysteines. Firstly, we observed that the LC:C88 has the highest level of free cysteine levels, among all the canonical intrachain disulfides, and across all the studied mAbs. Secondly, there are noticeable differences in free cysteine levels between germlines. IgG1λ mAbs display a consistently higher disulfide bond breakage compared to their IgG1κ counterparts, confirming earlier reports [14,29]. The susceptibility of a disulfide bond to reduction, under mild reducing conditions, is entirely dependent on the solvent accessibility of the participating cysteines and is independent of its free cysteine levels. Upon exposure to mild reducing environments, even for very short times, interchain and hinge disulfide bonds are reduced 100–1000 times faster compared to intrachain disulfide bonds. The solvent accessibility of cysteines estimated from molecular dynamics simulations of IgG1 shows that interchain and hinge cysteines have a 1000-fold higher solvent accessibility. 

Our work clearly establishes that the solvent accessibility of a cysteine is one of the key structural parameters that correlates with its rate of disulfide bond reduction. Ensuring minimal reductive stresses during manufacturing and storage is essential to ensure quality molecules with the desired efficacy and safety profiles. Antibody engineering to minimize solvent accessibility around cysteines could be an option to reduce the susceptibility of disulfide bonds. 

## Figures and Tables

**Table 1:** Comparison of cysteine-specific free thiol abundances from various studies and estimated susceptibilities in response to a reducing agent.

Disulfide Bond	Cysteine	Abundance (%) Native Condition		Susceptibility
		Native	Denatured	
Intrachain	HC:C22	20 ^a^ [10], <0.5 ^bc^ [14], 0.02–0.04 *	19.7 ^a^ [19], 0.37–0.62 ^#^	Low [14] Low [17] Low ^+^
	HC:C96	12 ^a^ [10], <0.5 ^bc^ [14], 0.11–0.25 *	23.1 ^a^ [19], 1.67–3.17 ^#^	
	HC:C147	6.5 ^a^ [10], 0.12–0.53 *	1.49–3.71 ^#^	
	HC:C203	2.4 ^a^ [10], 0.01–0.03 *	0.09–0.35 ^#^	
	HC:C370	6.6 ^a^ [10], 0.1–0.22 *	0.73–3.01 ^#^	
	HC:C428	5.3 ^a^ [10], 2–5 ^bc^ [16], 0.01–0.03 *	1.36–2.03 ^#^	
	LC:C23	67 ^bc^ [16], 0.15–1.0 *	1.61–4.95 ^#^	
	LC:C88	<1 ^a^ [10], 0.03–0.19 *	0.46–1.38 ^#^	
	LC:C134	<1 ^a^ [10], 0.01–0.17 *	0.56–2.12 ^#^	
	LC:C194	<1 ^a^ [10], 0.17–0.34 *	1.53–3.55 ^#^	
Interchain	LC:C214	1.5–2.6 ^bc^ [14], <2 ^bc^ [16], 0.09–0.68 *	0.72–4.78 ^#^	High [14] High ^+^
	HC:C223	1.5–2.6 ^bc^ [14], <2 ^bc^ [16]		
Hinge	HC:C229	<2 ^bc^ [16], 0.17–0.37 *	0.59–2.81 ^#^	High [14] High [17] High ^+^
	HC:C232	<2 ^bc^ [16], 0.16–0.39 *	0.37–3.20 ^#^	
SEFL	HC:C295	0.24–0.47 *	0.91–3.85 ^#^	intermediate ^+^
	HC:C305	0.07–0.09 *	0.27–1.68 ^#^	

* Range for both IgG1κ and IgG1λ (Figure S1), ^#^ range for both IgG1κ and IgG1λ (Figure 2b); ^+^ data from Figure 5b; ^a^ IgG1, ^b^ IgG1κ, and ^c^ IgG1λ.

## Data Availability

Data are contained within the article and Supplementary Materials.

## Supplementary Materials

The following supporting information can be downloaded at: https://www.mdpi.com/article/10.3390/antib12040083/s1, Table S1: Table showing the mapping between cysteine residues and the associated antibody domains for the four different mAbs used in our study. The cysteines have been numbered using their mature linear numbering. The corresponding IMGT [5] and Kabat [6] numbering for the variable domain, and the EU numbering [7] for the whole mAb are shown alongside. The linkages on the left indicate paired cysteines and * denotes that HC:C229 and HC:C232 are linked to their respective counterparts on the second chain. mAb1 and mAb2 contain κ light chains, while mAb3 and mAb4 contain λ light chains.; Table S2: Table showing the Pearson correlation coefficient for the rate of reduction (k) computed from peptide mapping data to the SASA values computed in molecular dynamics simulations. For all mAbs, we see an excellent linear correlation between k and SASA for all cysteine residues except for those in the hinges; Figure S1: Relative abundance of free cysteines tagged with NEM in native conditions. ND denotes residues for which the abundance was not measured; Figure S2: Distribution of S-S bond fluctuations in molecular dynamics simulations of IgG1κ and IgG1λ molecules (in duplicate, each 500 ns long), for the shown cysteine pairs. In each panel, the multiple thin lines correspond to the distributions computed for every 50 ns piece of the trajectories while the thick solid line denotes their average. We observed the S-S bond length to be normally distributed and the estimated variance ($\sigma^{2})$ was used to compute the associated spring constant as $\kappa_{ss}=\frac{k_{B}T}{\sigma^{2}}$, where $k_{B}$ is the Boltzmann constant and T is the absolute temperature, taken to be 300 K. The distribution of $\kappa_{SS}$ is shown in Figure 6a in the main manuscript; Figure S3: Schematic showing the disulfide bond between HC:C22 and HC:C96 in mAb1. Following Qin et al. [32] we quantify the orientations of the thiols group in terms of angles $\Theta_{1}$ and $\Theta_{2}$ computed from the positions of the CB and SG atoms in each cysteine; Figure S4: S-S bond angles $\Theta_{1}$ and $\Theta_{2}$ in molecular dynamics simulations of IgG1κ and IgG1λ molecules (in duplicate, each 500 ns long), for the shown cysteine pairs. The angles are defined in Supplementary Figure S3.

## References

[1] Tsumoto K., Isozaki Y., Yagami H., Tomita M. (2019). Future perspectives of therapeutic monoclonal antibodies. Immunotherapy.

[2] Glockshuber R., Schmidt T., Pluckthun A. (1992). The disulfide bonds in antibody variable domains: Effects on stability, folding in vitro, and functional expression in Escherichia coli. Biochemistry.

[3] Jacobsen F.W., Stevenson R., Li C., Salimi-Moosavi H., Liu L., Wen J., Luo Q., Daris K., Buck L., Miller S. (2017). Engineering an IgG Scaffold Lacking Effector Function with Optimized Developability. J. Biol. Chem..

[4] Liu L., Jacobsen F.W., Everds N., Zhuang Y., Yu Y.B., Li N., Clark D., Nguyen M.P., Fort M., Narayanan P. (2017). Biological Characterization of a Stable Effector Functionless (SEFL) Monoclonal Antibody Scaffold in Vitro. J. Biol. Chem..

[5] Lefranc M.P., Pommie C., Ruiz M., Giudicelli V., Foulquier E., Truong L., Thouvenin-Contet V., Lefranc G. (2003). IMGT unique numbering for immunoglobulin and T cell receptor variable domains and Ig superfamily V-like domains. Dev. Comp. Immunol..

[6] Kabat E.A., Wu T.T., Perry H., Gottesman K., Foeller C. (1991). Sequences of Proteins of Immunological Interest.

[7] Edelman G.M., Cunningham B.A., Gall W.E., Gottlieb P.D., Rutishauser U., Waxdal M.J. (1969). The covalent structure of an entire gammaG immunoglobulin molecule. Proc. Natl. Acad. Sci. USA.

[8] Lacy E.R., Baker M., Brigham-Burke M. (2008). Free sulfhydryl measurement as an indicator of antibody stability. Anal. Biochem..

[9] Moritz B., Stracke J.O. (2017). Assessment of disulfide and hinge modifications in monoclonal antibodies. Electrophoresis.

[10] Li X., Xiao L., Kochert B., Donnelly D.P., Gao X., Richardson D. (2021). Extended characterization of unpaired cysteines in an IgG1 monoclonal antibody by LC-MS analysis. Anal. Biochem..

[11] Chaderjian W.B., Chin E.T., Harris R.J., Etcheverry T.M. (2005). Effect of copper sulfate on performance of a serum-free CHO cell culture process and the level of free thiol in the recombinant antibody expressed. Biotechnol. Prog..

[12] Cao M., Wang C., Chung W.K., Motabar D., Wang J., Christian E., Lin S., Hunter A., Wang X., Liu D. (2018). Characterization and analysis of scFv-IgG bispecific antibody size variants. mAbs.

[13] Chung W.K., Russell B., Yang Y., Handlogten M., Hudak S., Cao M., Wang J., Robbins D., Ahuja S., Zhu M. (2017). Effects of antibody disulfide bond reduction on purification process performance and final drug substance stability. Biotechnol. Bioeng..

[14] Liu H., Chumsae C., Gaza-Bulseco G., Hurkmans K., Radziejewski C.H. (2010). Ranking the susceptibility of disulfide bonds in human IgG1 antibodies by reduction, differential alkylation, and LC-MS analysis. Anal. Chem..

[15] Xiang T., Chumsae C., Liu H. (2009). Localization and quantitation of free sulfhydryl in recombinant monoclonal antibodies by differential labeling with 12C and 13C iodoacetic acid and LC-MS analysis. Anal. Chem..

[16] Robotham A.C., Kelly J.F. (2019). Detection and quantification of free sulfhydryls in monoclonal antibodies using maleimide labeling and mass spectrometry. mAbs.

[17] Gurjar S.A., Wheeler J.X., Wadhwa M., Thorpe R., Kimber I., Derrick J.P., Dearman R.J., Metcalfe C. (2019). The impact of thioredoxin reduction of allosteric disulfide bonds on the therapeutic potential of monoclonal antibodies. J. Biol. Chem..

[18] Metcalfe C. (2022). A Review of Methodologies for the Detection, Quantitation, and Localization of Free Cysteine in Recombinant Proteins: A Focus on Therapeutic Monoclonal Antibodies. Front. Mol. Biosci..

[19] Zhang T., Zhang J., Hewitt D., Tran B., Gao X., Qiu Z.J., Tejada M., Gazzano-Santoro H., Kao Y.H. (2012). Identification and characterization of buried unpaired cysteines in a recombinant monoclonal IgG1 antibody. Anal. Chem..

[20] Wisniewski J.R., Zougman A., Nagaraj N., Mann M. (2009). Universal sample preparation method for proteome analysis. Nat. Methods.

[21] Zhang Z. (2009). Large-scale identification and quantification of covalent modifications in therapeutic proteins. Anal. Chem..

[22] MacLean B., Tomazela D.M., Shulman N., Chambers M., Finney G.L., Frewen B., Kern R., Tabb D.L., Liebler D.C., MacCoss M.J. (2010). Skyline: An open source document editor for creating and analyzing targeted proteomics experiments. Bioinformatics.

[23] Natesan R., Agrawal N.J. (2023). IgG1 and IgG4 antibodies sample initial structure dependent local conformational states and exhibit non-identical Fab dynamics. Sci. Rep..

[24] Eastman P., Swails J., Chodera J.D., McGibbon R.T., Zhao Y., Beauchamp K.A., Wang L.P., Simmonett A.C., Harrigan M.P., Stern C.D. (2017). OpenMM 7: Rapid development of high performance algorithms for molecular dynamics. PLoS Comput. Biol..

[25] Tian C., Kasavajhala K., Belfon K.A.A., Raguette L., Huang H., Migues A.N., Bickel J., Wang Y., Pincay J., Wu Q. (2020). ff19SB: Amino-Acid-Specific Protein Backbone Parameters Trained against Quantum Mechanics Energy Surfaces in Solution. J. Chem. Theory Comput..

[26] Hawkins G.D., Cramer C.J., Truhlar D.G. (1995). Pairwise solute descreening of solute charges from a dielectric medium. Chem. Phys. Lett..

[27] McGibbon R.T., Beauchamp K.A., Harrigan M.P., Klein C., Swails J.M., Hernandez C.X., Schwantes C.R., Wang L.P., Lane T.J., Pande V.S. (2015). MDTraj: A Modern Open Library for the Analysis of Molecular Dynamics Trajectories. Biophys. J..

[28] Shrake A., Rupley J.A. (1973). Environment and exposure to solvent of protein atoms. Lysozyme and insulin. J. Mol. Biol..

[29] Shen Y., Zeng L., Zhu A., Blanc T., Patel D., Pennello A., Bari A., Ng S., Persaud K., Kang Y.K. (2013). Removal of a C-terminal serine residue proximal to the inter-chain disulfide bond of a human IgG1 lambda light chain mediates enhanced antibody stability and antibody dependent cell-mediated cytotoxicity. mAbs.

[30] Kuninori T., Nishiyama J. (1985). Some properties of diastereomers formed in the reactions of N-Ethylmaleimide with biological thiols. Agric. Biol. Chern..

[31] Agrawal N.J., Dykstra A., Yang J., Yue H., Nguyen X., Kolvenbach C., Angell N. (2018). Prediction of the Hydrogen Peroxide-Induced Methionine Oxidation Propensity in Monoclonal Antibodies. J. Pharm. Sci..

[32] Qin M., Wang W., Thirumalai D. (2015). Protein folding guides disulfide bond formation. Proc. Natl. Acad. Sci. USA.

[33] Natesan R., Agrawal N.J. (2022). Non-covalent Fc-Fab interactions significantly alter internal dynamics of an IgG1 antibody. Sci. Rep..

[34] Saphire E.O., Parren P.W., Pantophlet R., Zwick M.B., Morris G.M., Rudd P.M., Dwek R.A., Stanfield R.L., Burton D.R., Wilson I.A. (2001). Crystal structure of a neutralizing human IGG against HIV-1: A template for vaccine design. Science.

[35] Song Y., Cai H., Tan Z., Mussa N., Li Z.J. (2022). Mechanistic insights into inter-chain disulfide bond reduction of IgG1 and IgG4 antibodies. Appl. Microbiol. Biotechnol..

