# Supplementary material for: Heterogeneity in Disulfide Bond Reduction in IgG1 Antibodies Is Governed by Solvent Accessibility of the Cysteines
Source: Antibodies (Basel). 2023 Dec 13;12(4):83. doi: 10.3390/antib12040083 (PMC10741012; doi:10.3390/antib12040083)
Supplement: Supplementary file 1 [file antibodies-12-00083-s001.zip › antibodies-2676159-supplementary.pdf]

Article

Heterogeneity in Disulfide Bond Reduction in IgG1 Antibodies Is Governed by Solvent Accessibility of the Cysteines

Ramakrishnan Natesan [1,†], Andrew B. Dykstra [2,†], Akash Banerjee [1] and Neeraj J. Agrawal [1,*]

[1] Amgen Inc., Process Development, 360 Binney St., Cambridge, MA 02141, USA; rnatesan@amgen.com (R.N.); abaner12@amgen.com (A.B.)
[2] Amgen Inc., Process Development, Thousand Oaks, CA 91320, USA; adykstra@amgen.com
[*] Correspondence: agrawaln@amgen.com; Tel.: +1-(617)-444-5503
[†] These authors contributed equally to this work.

Keywords: peptide mapping, differential alkylation, disulfide bond, molecular dynamics, SASA

Supplementary Information

Confidential Medical and Scientific Affairs

Table S1. Table showing the mapping between cysteine residues and the associated antibody domains for the four different mAbs used in our study. The cysteines have been numbered using their mature linear numbering. The corresponding IMGT [5] and Kabat [6] numbering for the variable domain, and the EU numbering [7] for the whole mAb are shown alongside. The linkages on the left indicate paired cysteines and * denotes that HC:C229 and HC:C232 are linked to their respective counterparts on the second chain. mAb1 and mAb2 contain κ light chains, while mAb3 and mAb4 contain λ light chains.

Cysteine residue numbering				Domain	Numbering			S-S bond type
mAb1(IgG1κ) (VK3/VH3)	mAb2(IgG1κ) (VK1/VH1)	mAb3(IgG1λ) (VL1/VH1)	mAb4(IgG1λ) (VL2/VH2)		IMGT	Kabat	EU	
LC:C23	LC:C23	LC:C22	LC:C22	V_L	23	23	23	intrachain
LC:C88	LC:C88	LC:C89	LC:C90	V_L	104	88	88	intrachain
LC:C134	LC:C134	LC:C138	LC:C138	C_L			134	intrachain
LC:C194	LC:C194	LC:C197	LC:C197	C_L			194	intrachain
LC:C214	LC:C214	LC:C215	LC:C215	C_L			214	interchain
HC:C223	HC:C220	HC:C231	HC:C231	C_H1			220	interchain
HC:C22	HC:C22	HC:C22	HC:C22	V_H	23	22	22	intrachain
HC:C96	HC:C96	HC:C96	HC:C97	V_H	104	92	96	intrachain
HC:C147	HC:C144	HC:C155	HC:C147	C_H1			147	intrachain
HC:C203	HC:C200	HC:C211	HC:C203	C_H1			200	intrachain
HC:C264	HC:C261	HC:C272	HC:C264	C_H2			261	intrachain
HC:C324	HC:C321	HC:C332	HC:C324	C_H2			321	intrachain
HC:C370	HC:C367	HC:C378	HC:C370	C_H3			367	intrachain
HC:C428	HC:C425	HC:C436	HC:C428	C_H3			425	intrachain
* HC:C229	HC:C226	HC:C237	HC:C229	Hinge			226	hinge-hinge
* HC:C232	HC:C229	HC:C240	HC:C232	Hinge			229	hinge-hinge
HC:C295	HC:C292	HC:C303	HC:C295	C_H2			-	intrachain
HC:C305	HC:C302	HC:C313	HC:C305	C_H2			-	intrachain

Confidential Medical and Scientific Affairs

Table S2. Table showing the Pearson correlation coefficient for the rate of reduction (k) computed from peptide mapping data to the SASA values computed in molecular dynamics simulations. For all mAbs, we see an excellent linear correlation between k and SASA for all cysteine residues except for those in the hinges.

Pearson r for k vs SASA	mAb1 (IgG1κ)	mAb2 (IgG1κ)	mAb3 (IgG1λ)	mAb4 (IgG1λ)
**Intrachain, interchain, hinge, SEFL**	0.56	0.74	0.87	0.87
**Intrachain, interchain, hinge**	0.67	0.87	1	1
**Intrachain, interchain, SEFL**	0.88	0.87	0.87	0.87
**Intrachain, interchain**	1	1	1	1

SI Figures

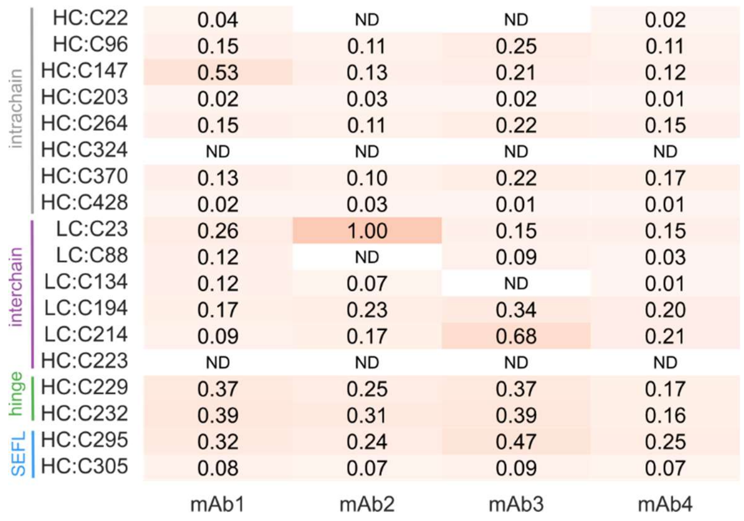

Figure S1. Relative abundance of free cysteines tagged with NEM in native conditions. ND denotes residues for which the abundance was not measured.

Confidential Medical and Scientific Affairs

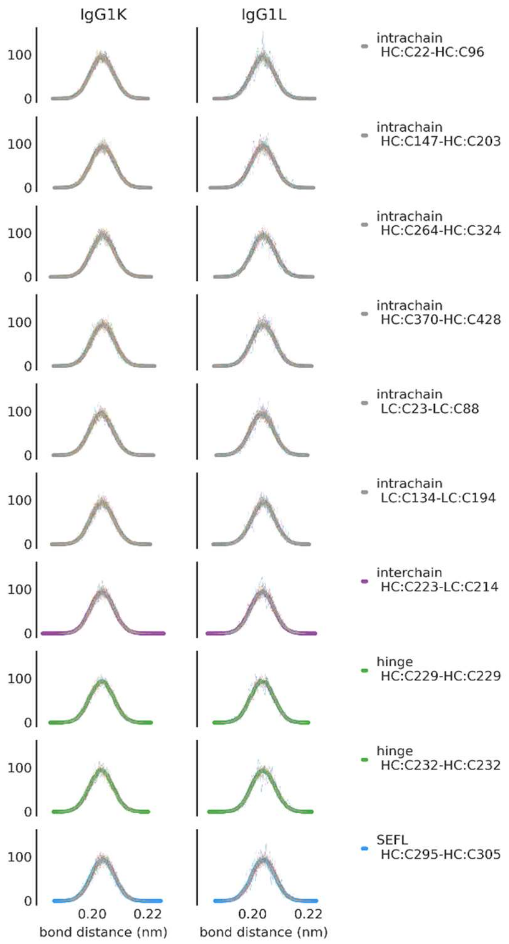

Figure S2. Distribution of S-S bond fluctuations in molecular dynamics simulations of IgG1κ and IgG1λ molecules (in duplicate, each 500 ns long), for the shown cysteine pairs. In each panel, the multiple thin lines correspond to the distributions computed for every 50 ns piece of the trajectories while the thick solid line denotes their average. We observed the S-S bond length to be normally distributed and the estimated variance (σ^2) was used to compute the associated spring constant as $\kappa_{SS} = \frac{k_B T}{\sigma^2}$, where k_B is the Boltzmann constant and T is the absolute temperature, taken to be 300K. The distribution of κ_{SS} is shown in Figure 6(a) in the main manuscript.

Confidential Medical and Scientific Affairs

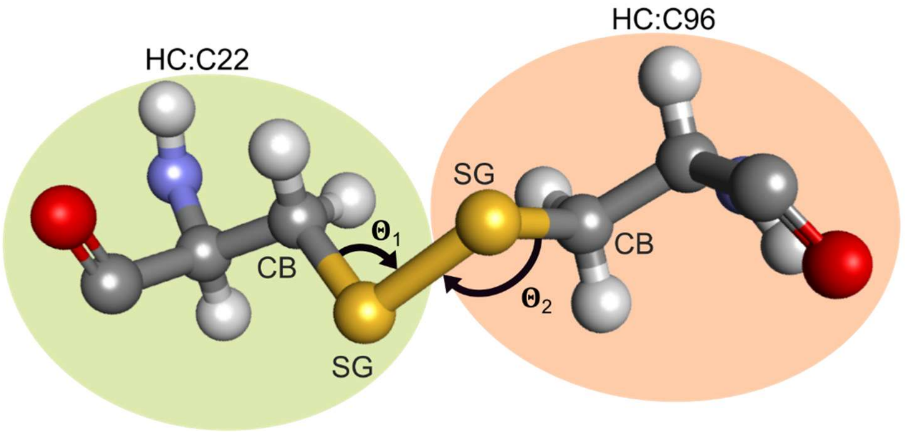

Figure S3. Schematic showing the disulfide bond between HC:C22 and HC:C96 in mAb1. Following Qin et. al. [32] we quantify the orientations of the thiols group in terms of angles Θ_1 and Θ_2 computed from the positions of the CB and SG atoms in each cysteine.

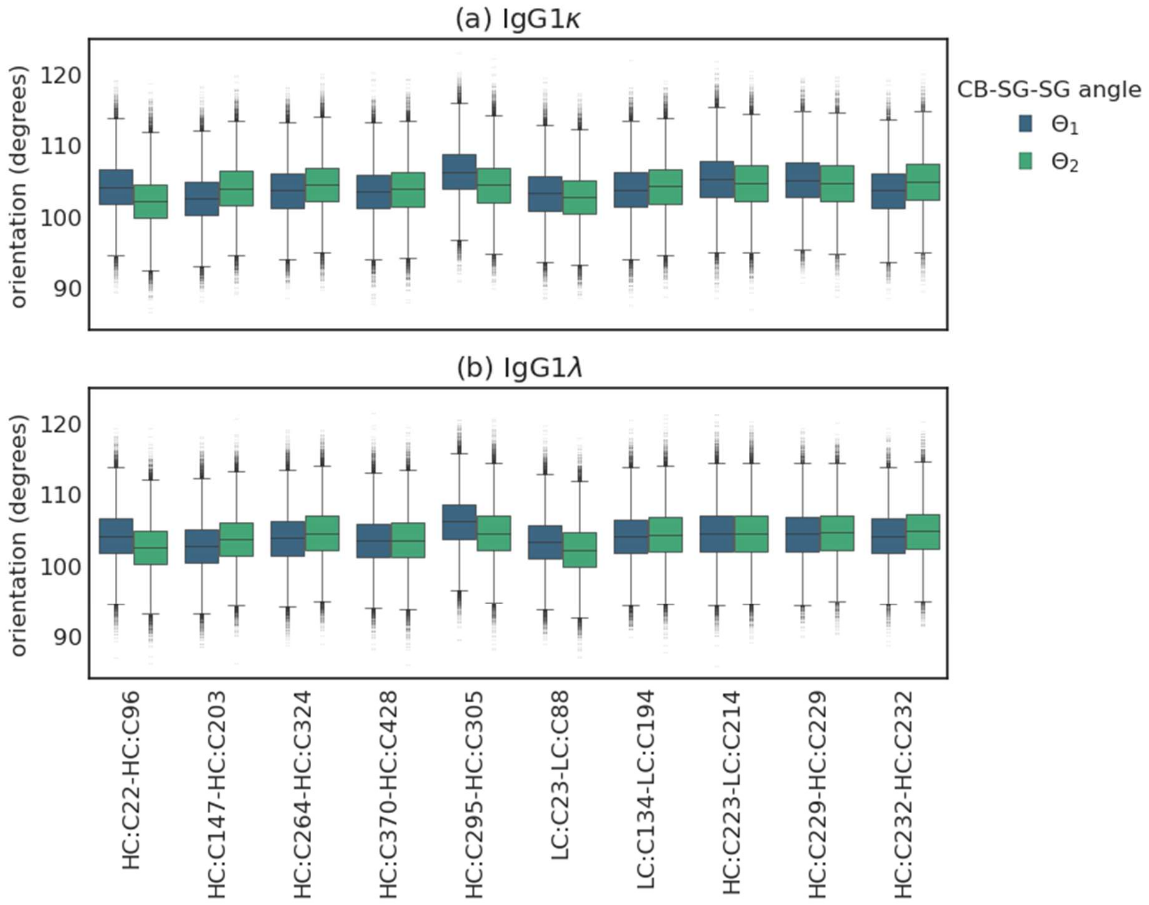

Figure S4. S-S bond angles Θ_1 and Θ_2 in molecular dynamics simulations of IgG1κ and IgG1λ molecules (in duplicate, each 500 ns long), for the shown cysteine pairs. The angles are defined in Supplementary Figure S3.
